# Application of Reactive Oxygen Species in Dental Treatment

**DOI:** 10.3390/jpm12091531

**Published:** 2022-09-18

**Authors:** Chiaki Komine, Satoshi Uchibori, Osamu Tsudukibashi, Yasuhisa Tsujimoto

**Affiliations:** 1 Department of Laboratory Medicine and Dentistry for the Compromised Patient, Nihon University School of Dentistry at Matsudo, Chiba 271-8587, Japan; 2 Department of Oral Function and Fixed Prothodontics, Nihon University School of Dentistry at Matsudo, Chiba 271-8587, Japan; 3 Department of Endodontics, Nihon University School of Dentistry at Matsudo, Chiba 271-8587, Japan

**Keywords:** reactive oxygen species, photolysis, antimicrobial photodynamic therapy, sterilization, smear layer removal, endodontic therapy

## Abstract

Reactive oxygen species (ROS) and free radicals, which have been implicated in inflammation, pain, carcinogenesis, and aging, are actually used in dental treatments such as tooth bleaching and composite resin polymerization. Recently, numerous studies have investigated the application of ROS in the medical and dental fields. In previous studies, ROS were generated intentionally through pathways such as photolysis, photocatalytic methods, and photodynamic therapy, which are used in the medical field to target cancer. In the field of dentistry, generated ROS are applied mainly for periodontal treatment and sterilization of the root canal, and its effectiveness as an antibacterial photodynamic therapy has been widely reported.. Given this background, the present article aimed to review the basic effects of ROS in dental medicine, especially endodontic therapy, and to discuss future applications of ROS.

## 1. Introduction

The terms “reactive oxygen species (ROS)” and “free radicals” are frequently used not only in medicine and dentistry, but also in other fields. However, for general clinical dentists, these terms are still unfamiliar and, thus, assumed to be poorly understood. Research on ROS and free radicals has made great strides, and their involvement in systemic diseases such as inflammation [1,2], pain [3,4], cancer [5,6,7], aging [7,8], adult diseases [9,10], and numerous other diseases of various organs [11,12] has been demonstrated. These include, of course, oral diseases such as periodontitis [13] and squamous cell carcinoma [14]. At the cellular level, ROS are also known to play important roles with respect to the second messengers and stem cell differentiation. That is, controlling ROS with antioxidants is expected to induce stem cells to differentiate into osteoblasts and contribute to bone regeneration [15]. Thus, ROS is an important and interesting element for living organisms in a variety of situations.

On the other hand, many novel methods for intentionally generating ROS and incorporating them into treatment in medical and dental medicine have also been studied. These methods, which generate ROS by irradiating photosensitizers (or photocatalysts) with appropriate excitation light, are known as photodynamic therapy (PDT) and antimicrobial photodynamic therapy (a-PDT), and are particularly effective against cancer [16,17,18,19] and bacteria-infected tissue [19,20,21], respectively.

The present review aims to examine ROS and free radicals by focusing on a-PDT in endodontic treatment, as well as the advantages and disadvantages of a-PDT and its potential for the future.

## 2. Materials and Methods

An electronic search was conducted for English language articles published between 1934 and 2021. Search terms for the general theory of endodontic therapy and ROS, and especially a-PDT and ROS in endodontic therapy, were entered into the following databases: PubMed, Ichushi-Web, and Google Scholar. The inclusion criteria were narrative reviews; in vitro, ex vivo, and clinical studies; and case reports written in English. The exclusion criteria were studies with conflicts of interest. The retrieved literature was identified for all titles, abstracts, and full texts. A total of 100 articles were included in this narrative review, including our previous papers. This paper provides a great deal of information on the application of ROS to dentistry, referring to the many findings reported to date, and provides researchers and practitioners with scientifically based knowledge.

## 3. What Are ROS and Free Radicals?

Oxygen comprises about 20% of the atmosphere. The oxygen breathed by humans is called triplet oxygen (^3^O_2_) in its stable (ground) state; it enters the body through respiration, and then binds to red blood cells, which allows for transport throughout the body. The concentration of ^3^O_2_ transported to peripheral tissues is low, about 1/150 of that in the atmosphere, and is taken up by cells. Then, ^3^O_2_ that has passed through the cell membrane undergoes various enzymatic reactions in the mitochondria to generate energy, after which, it acts as an oxidant, and the oxygen itself is reduced to eventually become H_2_O. When atoms, molecules, or ions lose electrons, they are defined as oxidized, and when they gain electrons, they are defined as reduced.

**Figure 1:**
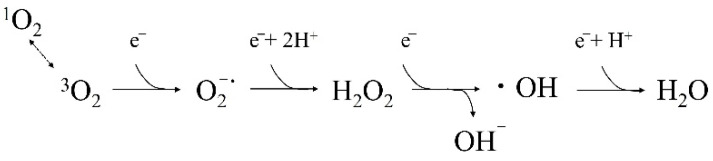
Redox reactions and reactive oxygen species (ROS) in vivo. Triplet oxygen (^3^O_2_) is reduced (one-electron reduction) and converted to superoxide anion (O_2_^−·^). The O_2_^−^ is then reduced by one more electron, and two H^+^ are added, leading to the formation of hydrogen peroxide (H_2_O_2_). When H_2_O_2_ is further reduced by one more electron, the bond between the O atoms can no longer exist stably, and the bond breaks to form the hydroxyl radical (^•^OH) and hydroxide ion (OH^−^). Finally, ^•^OH is reduced by one more electron, and one H^+^ is added, forming H_2_O. Singlet oxygen (^1^O_2_) is generated by an energy transition with ^3^O_2_.

ROS are the products of reactions that occur during the oxidation–reduction process of oxygen in cells (Figure 1). Unlike ^3^O_2_, superoxide anions (O_2_^−^), hydrogen peroxide (H_2_O_2_), hydroxyl radicals (^•^OH), and singlet oxygen (^1^O_2_), are highly reactive and commonly referred to as ROS. Radicals, on the other hand, are a general term for highly reactive and unstable substances with one or more “unpaired electrons.” When these radicals damage biological tissues, the tissues deprived of electrons become free radicals, and this process causes a chain reaction.

**Figure 2:**
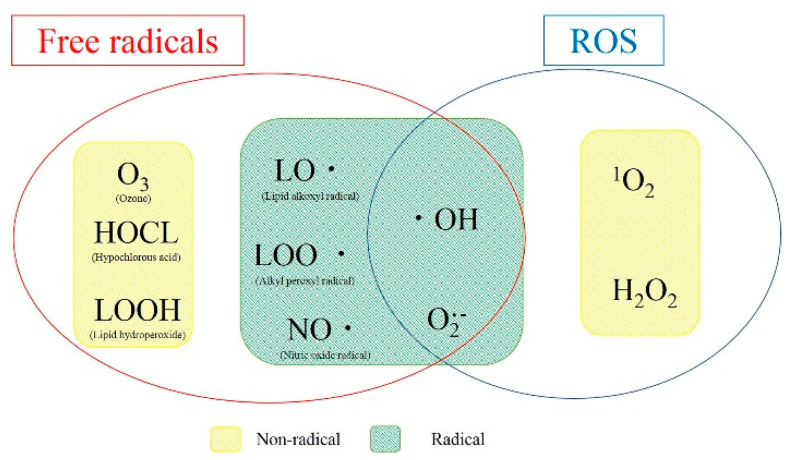
Diagram of radical and non-radical relationships in ROS and free radicals. Radicals are substances with unpaired electrons, and free radicals include non-radicals.

Figure 2 shows the relationship between ROS and free radicals, which are closely related to the organisms in which they occur. Therefore, radicals with unpaired electrons, ROS (radical or non-radical), and including the radical reserve, are widely defined as “free radicals”.

Among all ROS, O_2_^−·^ and ^•^OH are free radicals, and ^•^OH is known to be the most reactive [22]. ^•^OH is produced by the Fenton reaction [23] between transition metal ions such as Cu^2+^ and Fe^2+^ and H_2_O_2_, and by irradiation of H_2_O_2_ with light in the ultraviolet (UV) to blue light range [24]. Therefore, the ^•^OH produced by photolysis of H_2_O_2_ is applied in dentistry for sterilization [25,26,27,28,29,30,31] and tooth bleaching [32,33]. By contrast, ^1^O_2_ and H_2_O_2_ are classified as non-radical ROS. ^1^O_2_ is the most important factor in the mechanism of action of a-PDT, which is discussed below. Previous findings have shown that the mechanisms of a-PDT in endodontics are closely related to ROS, especially ^•^OH and ^1^O_2_.

## 4. Root Canal Cleaning by ROS

Endodontic treatment is the clinical management of bacterial disease, and the bacteria present in the root canal are the primary target of treatment [30,31]. However, the complexity of root canal structures makes complete debridement and bacterial elimination by instrumentation, irrigation, and intracanal medication virtually impossible [34]. In addition, another factor, the smear layer created during root canal shaping, seals the dentin tubules.

*Enterococcus faecalis* and *Candida albicans* are the species most frequently associated with refractory apical periodontitis [35]. *E. faecalis* has been reported to be naturally resistant to common disinfectants such as Ca (OH)_2_, iodine-containing tinctures, and antibiotics [36,37]. *C. albicans* is a fungus with a stronger cytoskeleton than bacteria, which makes it resistant to drugs [38], and its ability to adapt to various environments due to its dimorphic morphology reduces the success rate of root canal treatment [35,39]. Chemical disinfectants such as H_2_O_2_, ethylenediaminetetraacetic acid (EDTA), and sodium hypochlorite (NaClO) have conventionally been used for root canal irrigation for disinfection and removal of the smear layer [40,41]. However, conventional chemical disinfectants sometimes cause other problems such as tissue damage and accidents due to leakage [42,43]. Therefore, many researchers have been keenly interested in developing alternative sterilization methods with selective sterilizing power that do not cause damage to the organism.

**Figure 3:**
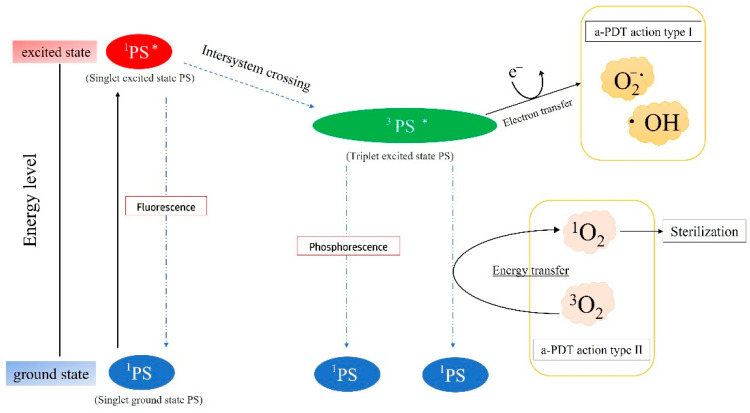
ROS and the mechanisms of photodynamic therapy (PDT; types 1 and 2). Singlet ground state photosensitizer (^1^PS) is irradiated with excitation light and changes to singlet excited state PS (^1^PS*). Subsequently, ^1^PS* changes to the triplet excited state PS (^3^PS*) or ^1^PS via fluorescence, luminescence, and intersystem crossing. The type 1 and 2 reactions occur by electron transfer to ^3^PS*, and by energy transition when ^3^PS* changes to ^1^PS, respectively.

Recently, various photochemically applied sterilization methods, especially a-PDT, have attracted increasing attention as alternative sterilization methods for bacterial oral diseases [19,20,44,45,46,47,48]. A-PDT requires three elements: ^3^O_2_, photosensitizing agents (PS), and excitation light. Then, the bacteria are effectively sterilized in the following manner via the a-PDT mechanism (a-PDT action type 2): (i) PS attach to the cell membrane of microorganisms, (ii) irradiation with light at a specific wavelength matched to the peak absorption of PS leads to the generation of ^1^O_2_, and (iii) bacterial death via destruction of the bacteria walls is induced by ^1^O_2_ [19,20,49,50] (Figure 3).

Despite the fact that the mechanisms of a-PDT are closely related to ROS, many studies have reported that the use of a-PDT for bactericidal effects requires the consideration of numerous variables when developing an a-PDT protocol, including light parameters, PS, and light delivery techniques [19,20,51,52,53,54]. Therefore, our laboratory used the electron spin resonance (ESR) spin-trapping method to supplement ROS and investigated the relationship between ROS generated by a-PDT and the bactericidal effect on microorganisms [55,56,57]. We also reviewed the findings of other researchers in regard to root canal cleaning with ^•^OH obtained by the photolysis reaction of H_2_O_2_ [25,26,27,28,29,30,31,58,59].

### 4.1. Bactericidal Efficacy for Bacteria Associated with Refractory Apical Periodontitis and the Appropriate PS Concentration for ^1^O_2_ Generation

**Figure 4:**
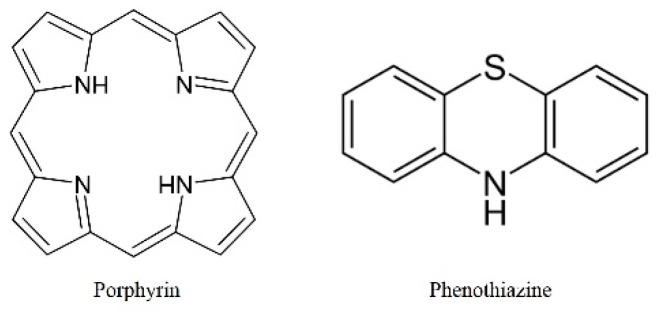
Typical structure of photosensitizing agents (PS). Photofrin® and 5-amiolevulinic acid are typical examples of porphyrin-based PS, while toluidine blue and methylene blue are typical examples of phenothiazine-based PS.

The bactericidal effect of a-PDT on refractory periodontitis-associated bacteria, such as *E. faecalis*, *C. albicans*, *Propionibacterium*, *Porphyromonas*, and *Prevotella* spp., has been reported in numerous papers, which suggests its efficacy even with different PS and light sources [19,20,44,45,46,47,48]. Although those previous reports were very significant, they were not uniform in terms of PS concentration or light source output. It is important to understand the minimum ^1^O_2_ required for a bactericidal effect to provide safer treatment. As the absorption of excitation light by PS triggers a series of mechanisms, there may be appropriate PS concentrations depending on the output of the excitation light. Many PS often use substances having the basic skeleton of porphyrins and phenothiazines [19,20], and many change color when exposed to solvent (Figure 4).

**Figure 5:**
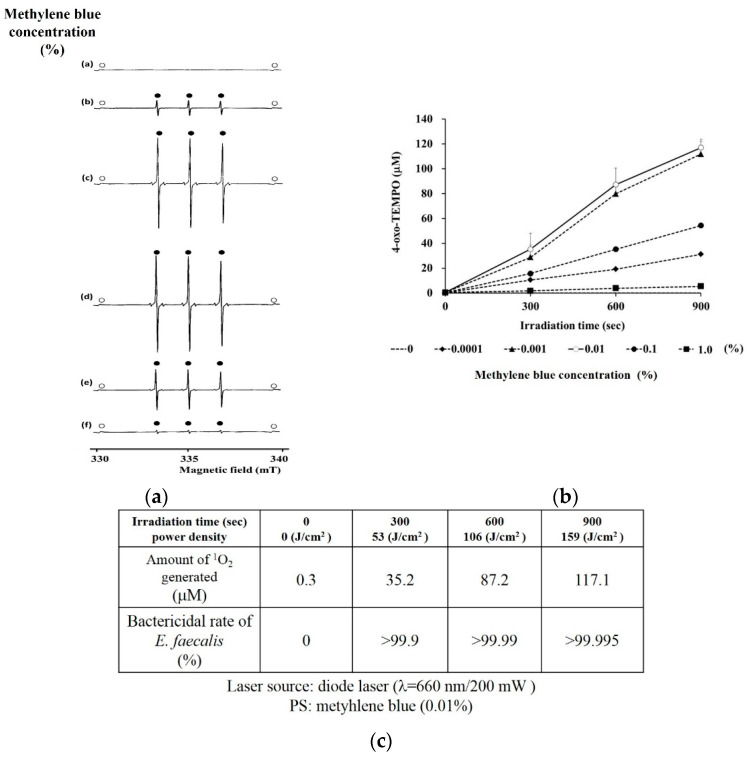
Relationship between the appropriate PS concentration and bactericidal effects in terms of ROS generation. (**a**) Measurement of ROS using an electron spin resonance (ESR) spin-trapping method. Typical ESR signal of 4-oxo-TEMPO generated by a diode laser (λ = 660 nm) with irradiation for each MB concentration (a: 0; b: 0.0001%; c: 0.001%; d: 0.01%; e: 0.1%; f: 1.0%). The 4-oxo-TEMP was used to trap the generated ^1^O_2_. The 4-oxo-TEMP traps ^1^O_2_ and converts it to 4-oxo-TEMPO. The white and black circles indicate the Mn^2+^ marker and the nitroxide radical, respectively. (**b**) Diode laser irradiation time and amount of ^1^O_2_ generated. ^1^O_2_ occurred at all MB concentrations in a laser irradiation time-dependent manner. The largest amount of ^1^O_2_ generated was at 0.01% MB. (**c**) Relationship between the amount of ^1^O_2_ generated and the bactericidal effect of *Enterococcus faecalis*. Adapted from Ref. [55].

Therefore, the irradiation of excitation light should not be considered to produce much ^1^O_2_ in either case or to have a bactericidal effect because low PS concentrations result in little PS excitation, whereas high PS concentrations result in no excitation light penetrating the PS. Actually, in our previous report [55] using a diode laser (660 nm, 200 mW) and methylene blue (MB) as the light source and PS, respectively, we were able to determine the appropriate concentration of MB to generate ^1^O_2_. Figure 5a shows the typical ESR-spectra of 4-oxo-TEMPO, which is the ^1^O_2_-specific oxidation from 4-oxo-TMP to 4-oxo-TEMPO, and Figure 5b shows the amount of ^1^O_2_ generated from each concentration of excited MB. The amount of generated ^1^O_2_ increased in the following order: 0.01% > 0.001% > 0.1% > 0.0001% > 1.0%. The most efficient generation of a large amount of ^1^O_2_ was from 0.01% excited MB, which is the appropriate PS concentration for this output. Further, the amount of generated ^1^O_2_ necessary to kill *E. faecalis* (>99.9%) was at least 35.2 μM (Figure 5c).

Tanaka et al. [60] used a diode laser (λ = 664 nm, 20 J/cm^2^) as a light source and compared MB concentrations of 1, 5, 20, 100, and 400 μM. When a-PDT was applied to the methicillin-resistant *Staphylococcus aureus* bactericidal test, a biphasic dose response was observed with the highest bactericidal effect at 100 μM and a lower effect at 1 or 400 μM. In addition, Chan et al. [61] observed a sterilization effect when a diode laser (λ = 665 nm, 21.2 J/cm^2^) with 0.01% MB was used to irradiate oral bacteria. Therefore, it is considered that 0.001%–0.01% MB on a-PDT corresponds precisely to the appropriate PS concentration of MB, which is between 26.7 and 267 μM, and that the bactericidal effect is due to the efficient generation of ^1^O_2_. However, these reports also differ in terms of irradiation conditions, such as the irradiation distance or width; therefore, irradiation conditions should be standardized for clinical applications in the future. The appropriate concentration for PS other than MB from the perspective of ^1^O_2_ generation also needs to be considered.

### 4.2. Sterilization Mechanism of a-PDT by ^1^O_2_

Considering bactericidal action and ^1^O_2_, Allen et al. [62] first reported that neutrophils may produce ^1^O_2_ via the catalysis of myeloperoxidase during respiratory bursts. Subsequently, Krinsky [63] suggested that neutrophil-derived ^1^O_2_ mediates bacterial killing. Further, Tatsuzawa et al. [64] suggested that phagocytic leukocytes produce ^1^O_2_ as a major bactericidal oxidant in the phagosome, and may also interact with the cytoplasmic membrane of microorganisms, especially *E. coli* and other Gram-negative bacteria, and damage respiratory enzymes while inhibiting ATP formation. That is, the small amount of ^1^O_2_ generated in vivo is not toxic to eukaryotic cells. Due to its short half-life (10^−6^ s), ^1^O_2_ only inactivates enzyme activity on the surface of nearby prokaryotic cells. In the a-PDT mechanism, on the other hand, ^1^O_2_ generated by PS and excitation light from outside the cell destroys the cell wall and/or membrane, and then PS is transferred into the cell. Next, ^1^O_2_ generated by photoexcitation destroys intracellular organelles, leading to cell death. A large amount of ^1^O_2_ is produced by PS excitation, and, unlike in vivo, the amount of ^1^O_2_ produced increases with the irradiation time of the excitation light. Additionally, the ^1^O_2_ produced is a nonspecific oxidant for which there is no defense [65,66]. Importantly, the amount of ^1^O_2_ used for treatment should be minimized to help prevent excessive oxidative damage to normal cells [67].

**Figure 6:**
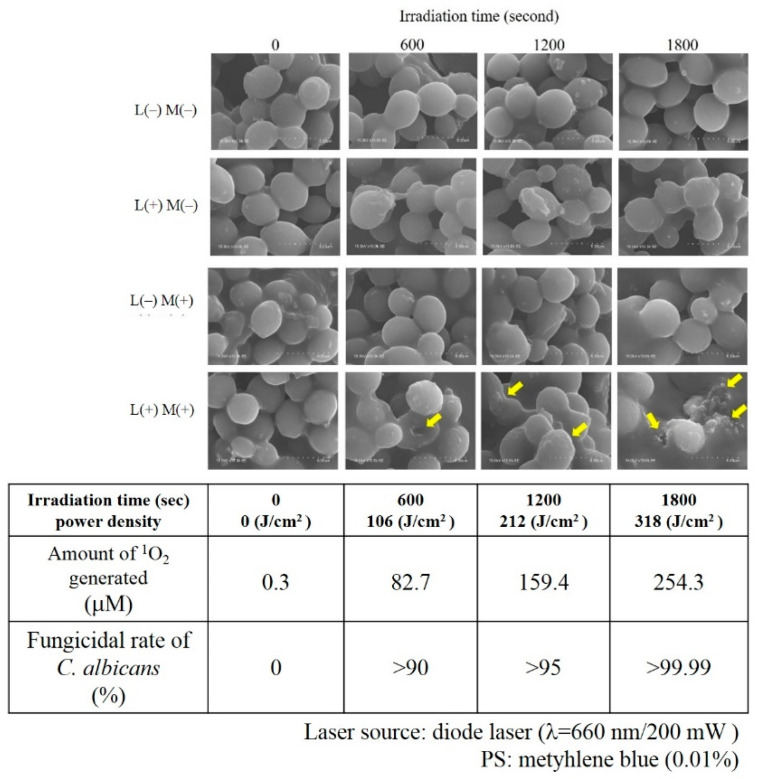
Morphological evaluation of *Candida albicans* during ^1^O_2_ exposure in scanning electron microscopy. A previous report designated *C. albicans* as follows: with diode laser irradiation: L(+); without diode laser irradiation: L(−); with MB: M(+); without MB: M(−). These were combined to form four groups: L(+)M(+); L(+)M(−); L(−)M(+); and L(−)M(−). Only L(+)M(+) showed changes in *C. albicans* morphology. The yellow arrows indicate that the morphology of *C. albicans* was damaged by ^1^O_2_, fused, and underwent further morphological disruption, resulting in an amorphous lump. The fungicidal effect of *C. albicans* was about 82.7, 159.4, and 254.3 μM with ^1^O_2_ generation in >90%, >95%, and >99.99%, respectively. Reprinted/adapted with permission from Ref. [56].

Therefore, our laboratory used scanning electron microscopy (SEM) to evaluate visually the mechanism of ^1^O_2_ in the fungicidal action of *C. albicans*, a eukaryote with a cell wall, and further examined the amount of ^1^O_2_ produced along with its fungicidal effect [56]. The results of a-PDT against *C. albicans* using 0.01% MB as PS and a diode laser (660 nm, 200 mW) as the light source showed that at least 245.3 μM of ^1^O_2_ was required to achieve >99.99% fungicidal activity. That is, compared with bacteria, which are prokaryotic cells, *C. albicans* requires a very large amount of ^1^O_2_. The reason for this may be attributed to the differences in the cytoskeleton mentioned previously. Figure 6 shows irradiation time-dependent fusion, i.e., fusion that is dependent on the amount of ^1^O_2_ generated, as well as the loss of the normal morphology of single independent cells as they begin to fuse with each other. In SEM images exposed to 245.3 μM of ^1^O_2_, the fused cells further lose their morphology and become amorphous lumps (Figure 6).

**Figure 7:**
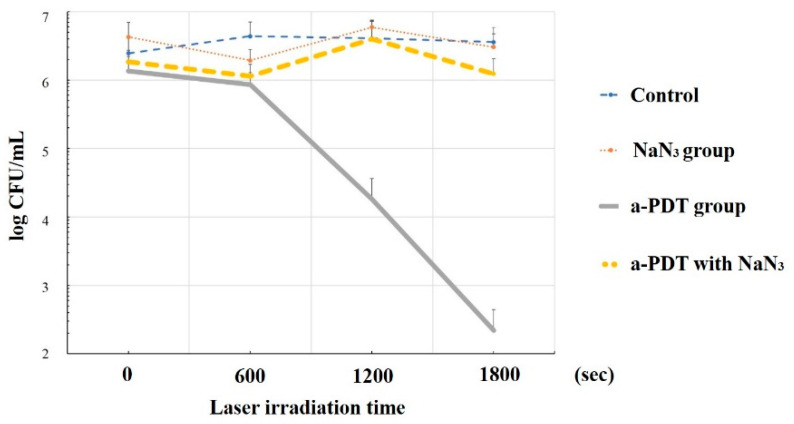
Fungicidal effect of antimicrobial PDT (a-PDT) in ^1^O_2_ scavenging. A-PDT (same conditions as in Ref. [56]) showed reduced bacterial counts of more than 3-log but no reduction at all under the sodium azide (NaN_3_) combination. Reprinted/adapted with permission from Ref. [57], 2019, Nihon university society of oral science.

Conversely, when sodium azide (NaN_3_, 10 mM), which is a specific scavenger of ^1^O_2_ and at a concentration not toxic to *C. albicans*, was added to the MB solution and a-PDT was performed on *C. albicans* under the same conditions, no fungicidal effect was observed (Figure 7), which suggests that exposure to large amounts of ^1^O_2_ affects not only the target microorganisms but also the host, which is a eukaryotic cell [57].

In summary, our previous reports suggest that a large amount of ^1^O_2_ is required to disinfect *C. albicans* by a-PDT, and that the mechanism underlying the a-PDT’s bactericidal effects on microorganisms is the physical injury caused by ^1^O_2_ [56,57].

### 4.3. Bactericidal Effect of ^•^OH Resulting from Other Photochemical Reactions

**Figure 8:**
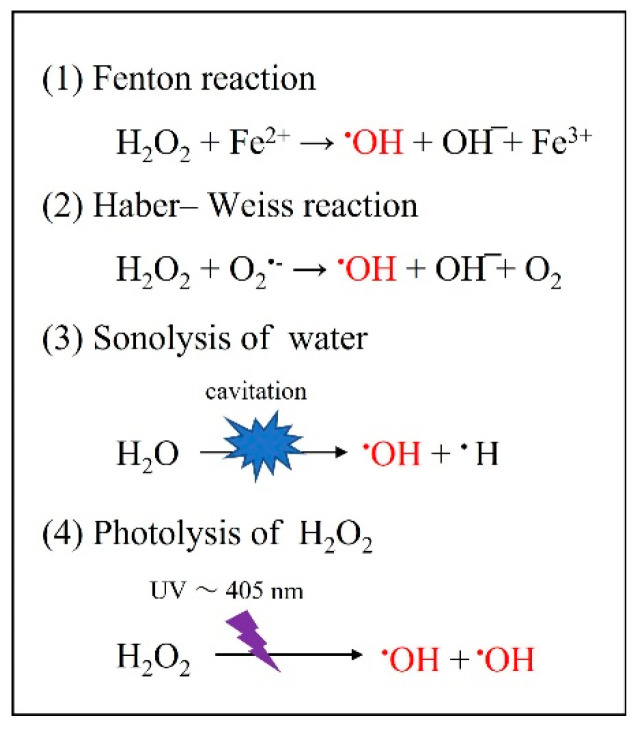
The processes of •OH production.

^•^OH is characterized by having the shortest half-life (10^−8^ s) and the strongest oxidizing power among all ROS [22]. The ^•^OH formation systems include (1) the Fenton reaction [23], (2) the Haber–Weiss reaction [68], (3) the sonolysis of water [69], and (4) the photolysis of H_2_O_2_ [25,26,27,28,29,30,31,58,59] (Figure 8).

In these systems, ^•^OH generated by the photolysis of H_2_O_2_ in reactive systems has been applied to oral bacterial infections, such as caries [25,70,71], periodontal disease [25,27,72], periapical disease [25,71], and aspiration pneumonia [25,26,29,59]. The wavelength spectrum used for the photolysis reaction of H_2_O_2_ ranges from UV to blue light, which is visible light [24]. Therefore, many reports in the dental field use a wavelength of 405 nm to generate ^•^OH [25,26,27,28,29,30,31,58,59,70,71].

The research group of Kanno and Sasaki is a pioneer in the use of this photolysis reaction for sterilization by ^•^OH and the effect of ^•^OH on dental materials [25,26,27,28,29,30,69,70,71,72,73,74,75]. In particular, they reported that an ^•^OH generation rate of approximately 200–300 μM is required to achieve a 4-log reduction in the number of bacteria, i.e., staphylococcus aureus infection (*Staphylococcus aureus*), dental caries (*Streptococcus mutans*), periodontal disease (*Aggregatibacter actinomycetemcomitans*), and refractory apical periodontitis (*E. faecalis*) [25]. The fact that this disinfection method is possible with 3% H_2_O_2_, which is routinely used in dental practice, and 405 nm light-emitting diode (LED), which is used for tooth bleaching, is expected to lead to further developments.

### 4.4. Effect of ^•^OH on Smear Layer Removal

**Figure 9:**
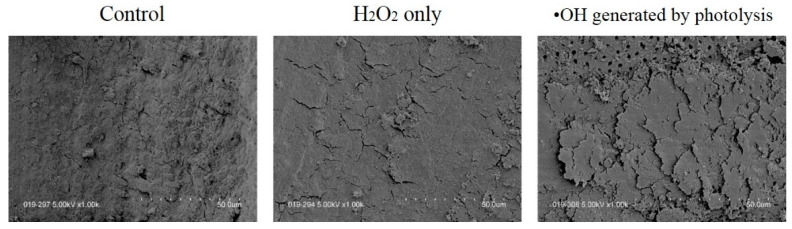
Comparison of typical scanning electron microscopy images of root canal surfaces. The control sample is entirely covered by a smear layer, and H_2_O_2_ alone causes a smear layer similar to fish scales but no dentin tubules are observed. In photolysis, more smear layers resembling fish scales are observed, and dentin tubules are clearly visible. Reprinted/adapted with permission from Ref. [58], 2021, the Quintessence.

The use of instruments such as reamers, files, and bars can result in the formation of a smear layer on the dentin surface [76]. This smear layer contains dentin components, remnants of the periodontal ligament formation process, pulp tissue, and bacteria [77], and, thus, may interfere with the penetration of the irrigation solution and cause leakage between the root canal wall and filling material [78]. Therefore, our laboratory was interested in the strong oxidizing power of ^•^OH, so we investigated not only its bactericidal effect but also its effect on the removal of the smear layer. First, we examined whether ^•^OH generated by the Fenton reaction of metal ions with 3% H_2_O_2_ could remove the smear layer [79,80,81]. The results showed that H_2_O_2_ alone was not effective for removing the smear layer. Although the smear layer was not removed when ^•^OH was generated, it was confirmed that the surface of the smear layer was roughened. In other words, it was assumed that ^•^OH would damage the inorganic and organic materials that formed the smear layer, and decompose and then clean the smear layer while roughening it. Therefore, the generation and exposure of more ^•^OH in the root canal raised expectations for the removal of the smear layer. Next, ^•^OH produced by a photolysis reaction using 3% H_2_O_2_ and a 405 nm LED was used to remove the smear layer [58]. The amount of ^•^OH generated by the photolysis reaction was significantly higher than that generated by the Fenton reaction. Although a large amount of ^•^OH is dangerous for living organisms, we consider it to be safe in a closed space only inside the root canal, where the light can reach H_2_O_2_. The results showed that when the H_2_O_2_ was irradiated with 405 nm of LED light for 3 min, the smear layer was not completely removed but was roughened or partially removed, and some dentin tubules could be observed (Figure 9).

Under this irradiation condition, it is calculated that about 510 μM equivalent of ^•^OH is generated. The effect of about 510 μM ^•^OH on the smear layer was comparable to that of alternating washes with 5% NaClO or 3% H_2_O_2_ [82] and immersion in 1% EDTA for 2 min [83].

Based on the above, ^•^OH is an important factor in the removal of the smear layer. Further, Tsujimoto et al. [82] reported that to remove the smear layer more efficiently, it is important to reflux instead of immerse the chemical solution. Therefore, our laboratory is now examining the effect of smear layer removal by generating ^•^OH under different immersion and reflux conditions. In addition, the possibility of smear layer removal by ^1^O_2_ is also currently under investigation. If an effective smear layer removal method using ^•^OH and ^1^O_2_ can be established, it may become an alternative cleaning method to NaClO and EDTA. This section may be divided by subheadings. It should provide a concise and precise description of the experimental results, their interpretation, as well as the experimental conclusions that can be drawn.

## 5. Discussion

**Figure 10:**
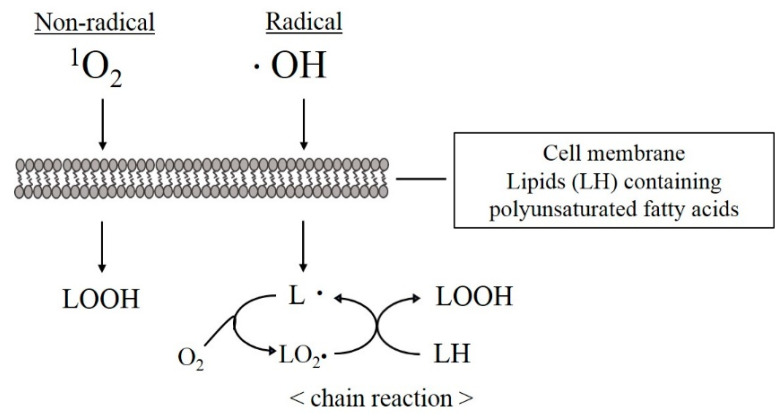
Differential effects of radical and non-radical reactions. Non-radicals are stoichiometric reactions that oxidize the target molecule and terminate the reaction, whereas radicals oxidize the target molecule and generate new radicals at the same time, and the reaction continues repeatedly.

Numerous papers have demonstrated that ROS can play an active role in root canal sterilization and smear layer removal. The traditional purpose of applying ROS is to eliminate the unexpected side effects caused by the use of NaClO or EDTA, with an emphasis on safety. Therefore, several points must be considered when applying ROS, such as the difference in post-action effects between the radical ^•^OH and the non-radical ^1^O_2_. These basic actions occur by directly injuring the substance from hydrogen atom transfer through the oxidizing powers. In general, radicals induce chain reactions [84], whereas non-radicals are quantitative in character [85], so the subsequent effects should be considered greater for the radicals (Figure 10).

In vivo, cells have antioxidant enzymes such as superoxide dismutase and catalase [86,87], and, furthermore, cell membranes damaged by radicals can be stopped in a radical-induced chain reaction by antioxidants such as tocopherol and ascorbic acid [15,88,89]. In root canals, however, there are no antioxidant enzymes or antioxidants to stop the radical-induced chain reaction that damages the organic components of dentin. As a result, even if the objectives of sterilization and smear layer removal are achieved, subsequent dental treatment, i.e., mechanical properties of dentin and bond strength of composite resins, may be adversely affected. Abe et al. [90] reported a significant decrease in bond strength between orthodontic appliances and enamel immediately after tooth bleaching methods involving ^•^OH. Therefore, the effect of composite resin on the bond strength to dentin after ROS exposure should also be examined. Conversely, radicals act on the cell membranes of microorganisms, which triggers a chain reaction that may be effective against colonizing bacterial populations. Furthermore, we believe that ROS are particularly effective because anaerobic bacteria have few antioxidant enzymes. Although these ideas are still speculative, they should be considered in the future when using ROS. It should also be noted that ROS inflict nonselective injury on adjacent substances. The optimal application of a-PDT is to let targets take up PS and then selectively kill the targets, similar to PDT in cancer therapy. The selective killing of microorganisms is essential for the future development of a-PDT. In addition, in the case of ^•^OH disinfection using the photolysis method, safety considerations such as the use of antioxidants (tocopherol [88], ascorbic acid [89], and catechin [91]) to scavenge radicals and prevent post-action chain reactions are also considered necessary. The control of ROS and free radicals generated in the root canal actually involves not only the a-PDT and photolysis methods but also NaClO, which has an intramolecular hypochlorite radical (OCl^−^) and is routinely used by clinicians. Although it is no longer the gold standard, our laboratory has shown that alternating irrigation with H_2_O_2_ generates ROS and free radicals such as O_2_^−·^, ^•^OH, and 5,5-dimethyl-1-pyrrolidone-(2)-oxyl-(1) (DMPO-X) radical [92,93].

While many dental treatments using ROS have been performed in the past, dental clinicians still tend to have limited knowledge of ROS. ROS and free radicals are associated with inflammation, carcinogenesis, and aging and can not only cause injury to living organisms but also kill microorganisms and viruses depending on how they are applied. On the other hands, various disinfection methods for the application of ROS have not yet standardized experimental conditions, and many have concluded that its bactericidal effect should be used in combination with conventional cleaning by NaClO and EDTA. In other words, it must be noted that there is a limit to sterilization by ROS alone. A further weakness is that this review is a narrative review, not a systematic review, as it does not analyze the data or study group size to determine the validity of the results.

Research on ROS, such as bleaching and disinfection methods, using titanium dioxide as a photocatalyst [94,95] and photofunctionalization of implants [95], is also developing. Furthermore, photochemistry has recently been applied for the detection of oral cancer [96,97,98,99] and tooth decay [100]. Additional findings are expected to be investigated and the development of dental treatment using ROS is expected to continue in the future.

## 6. Conclusions

The following four points are required for the general clinical application of a-PDT or photolysis methods:

1. The light source and PS should be standardized, taking into account the optimal generation of ROS.
2. The possible disadvantages of dental treatment after ROS exposure should be considered.
3. The advantages and disadvantages to the organism induced by ROS should be familiarized.
4. A-PDT should be used as an adjunct to conventional disinfection methods at this time, and when using NaClO together with a-PDT, it might be reducing the concentration of NaClO, thereby reducing harmful effects on living organisms.

## Data Availability

The data used to support the findings of this study are included within the article and are available from the corresponding author upon reasonable request.
